# Ultra-Compact, High-Efficiency Vertical Meta-Grating Couplers for Meta-Photonic Integrated Circuits

**DOI:** 10.3390/nano15080583

**Published:** 2025-04-11

**Authors:** Hang Cheng, Jiagui Wu, Yue Wang, Chongchong Ran, Haitang Li, Yu Wang, Yuanhui Li, Sen Zhang, Chunhui Wang, Junbo Yang

**Affiliations:** 1 National Key Laboratory of Laser Spatial Information, Harbin Institute of Technology, Harbin 150001, China; hiteechh@163.com (H.C.); seraph@hit.edu.cn (Y.W.); yvonne@hit.edu.cn (Y.W.); 23b921026@stu.hit.edu.cn (Y.L.); 2 College of Science, National University of Defense Technology, Changsha 410073, China; 201974010228@nwnu.edu.cn (C.R.); lht15223540254@163.com (H.L.); zhangsen@nudt.edu.cn (S.Z.); 3 School of Physical Science and Technology, Southwest University, Chongqing 400715, China; mgh@swu.edu.cn

**Keywords:** ultra-compact, high efficiency, vertical meta-grating couplers, meta-photonic integrated circuits

## Abstract

Vertical meta-grating couplers (VMGCs), while essential for flexible spatial beam coupling in meta-photonic integrated circuits (MPICs), suffer from inherently low coupling efficiency that hinders broader applications. In this study, we introduce an improved adjoint optimization method with high computational efficiency and excellent optimization effectiveness. Utilizing this method, we demonstrate an ultra-compact single-polarization VMGC achieving 81.57% coupling efficiency with a 92 nm 3 dB bandwidth, and a dual-polarization beam-splitting coupler with over 52% coupling efficiency for both polarizations, a 3 dB bandwidth exceeding 60 nm, an ultra-high extinction ratio of over 26.4 dB, and negligible polarization dependent loss at 1550 nm. To the best of our knowledge, this achievement represents the best simulation record to date for a perfect vertical coupler without bottom reflectors.

## 1. Introduction

Grating couplers are essential optical interfaces widely employed in photonic devices for applications such as lidar [1,2], optical communication [3], and far-field imaging and sensing [4,5], owing to their flexible layout, straightforward optical path design, high integration potential, and broadband capabilities [6]. Vertical grating couplers, in particular, mitigate stringent light source positioning requirements, facilitating compact photonic integrated circuits and enabling ultra-compact systems, which is especially vital for large-scale transceiver antenna arrays in photonic integrated lidar applications [2,7].

However, perfectly vertical grating couplers are susceptible to strong back-reflections, which results in lower coupling efficiencies. Therefore, the design of vertical grating couplers is undoubtedly complicated. To minimize optical transmission losses, routing waveguides in photonic integrated circuits typically possess a width of approximately 0.5 μm [8]. Thus, a tapered mode converter is necessary for connecting the vertical grating coupler to the routing waveguide (except for focused grating couplers). Achieving effective low-loss adiabatic conversion mandates a sufficiently long waveguide taper—often several hundred micrometers long for a standard 10 μm grating coupler transitioning to a 0.5 μm wide single-mode waveguide. This requirement presents a significant hurdle to maximizing the integration potential of grating couplers. As for the focusing grating coupler, although it has a smaller size due to the absence of a taper, the curvature of its grating is difficult to precisely control during the fabrication process, which significantly degrades its performance. The utilization of bottom reflectors provides a promising route to achieving compact and high-performance vertical grating couplers [9,10]. Nevertheless, the inherent complexity of the multi-layered heterogeneous structure imposes substantial limitations on its practical application. Double-layer grating couplers, which theoretically offer near-100% directivity and minimal back-reflection [11], have attracted significant attention. However, practical reports and performance demonstrations remain limited [12]. Some VMGCs designed by neural networks have also been reported, but the coupling efficiency of such devices is only around −2 dB [13,14].

In recent years, the emergence of meta-materials and the application of inverse design methods have significantly enhanced the design freedom in photonic devices, resulting in various unprecedented ultra-compact and high-performance meta-photonic devices [15,16]. Notably, meta-grating couplers transcend conventional periodic structures by achieving complex spatial configurations with subwavelength precision, making the development of ultra-compact, high-performance couplers feasible [17]. Fortunately, this type of device can be realized by various inverse design methods [18,19]. Among these, adjoint (ADJ) optimization was prioritized for its capacity to accommodate a high degree of design freedom, its computational efficiency (1 forward simulation and 1 adjoint simulation per iteration vs. 10^4^–10^5^ for metaheuristics), and its deterministic gradient guidance, thereby avoiding the ‘black-box’ limitations of neural networks. While deep learning can accelerate parameter exploration, its demand for massive training data (>10^4^ labeled designs) is impractical for high-dimensional photonic problems.

This study presents an improved optimization method for the VMGC. Utilizing this approach, we designed two ultra-compact VMGCs.The first, optimized for single polarization, achieves a coupling efficiency of 81.57% with a broad 3 dB bandwidth of 92 nm. The second, designed for dual-polarization splitting, demonstrates coupling efficiencies of 52.82% and 53.11% at 1550 nm for TE- and TM-polarized light, respectively, with a 3 dB bandwidth exceeding 60 nm. Furthermore, this dual-polarization device exhibits a polarization-dependent loss (PDL) of less than 0.42 dB at 1550 nm across incident polarization angles ranging from 0° to 180°. We also investigated the influence of etching depth, incident angle, initial structure, materials, and processing technology on the optimization performance of the single-layer VMGC. These findings provide valuable insights for the design of analogous devices. The proposed optimization method has broad applicability to diverse meta-photonic devices, and the designed couplers are particularly well suited for meta-photonic integrated circuits, offering the potential for applications in photonic integrated circuit lidar, optical communications, optical imaging, inverse design of photonic devices, and others.

## 2. Design and Simulation

### 2.1. Simulation Settings

**Figure 1:**
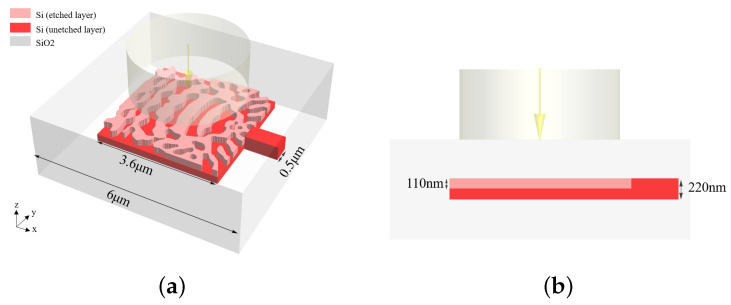
Schematic diagram of the VMGC: (**a**) full 3D structure. (**b**) Front view.

This study focuses on a silicon-on-insulator (SOI) platform with a 220 nm silicon layer atop a 2 μm buried oxide (BOX). This platform is widely adopted for CMOS-compatible photonic integrated circuits due to its high index contrast and compatibility with foundry processes. Here, a single-polarization VMGC is used as an example to describe its simulation settings, as shown in Figure 1. The optimized region of the device is a square with an edge length of 3.6 μm. In particular, the etching depth of the grating is 110 nm. A single-mode waveguide with a cross-section of 500 nm × 220 nm is placed on the right side of the design region as the output port. We set the 3D FDTD region and the minimum grid pixel size to 6 μm × 6 μm × 3.4 μm and 20 nm, respectively, to ensure the validity and correctness of the simulation. The effective refractive index of Si and SiO2 is 3.47668 and 1.44401, respectively. In order to maximize the coupling of light source energy within the size of existing devices, we set the mode field diameter (MFD) to 2.08 μm. The coupling efficiency of each grating coupler is defined as the ratio of the power coupled into the fundamental mode of the output silicon waveguide to the incident power of the Gaussian beam source, which serves as a suitable approximation of the mode profile emitted by a single-mode fiber (SMF).

### 2.2. Optimization Method

**Figure 2:**
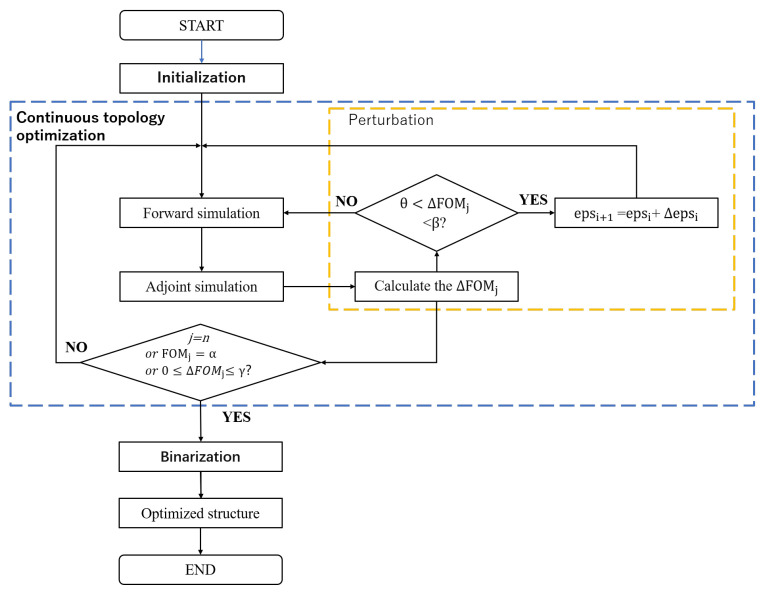
The flowchart of the improved ADJ optimization method.

We designed the VMGCs by using our improved adjoint optimization method combined with the finite-difference time-domain solver (Ansys Lumerical FDTD). The optimization objective (also known as the figure of merit, FOM), defined as maximizing the overlap integral between the fundamental mode of the output waveguide and the incident Gaussian source at λ = 1550 nm, was achieved through a three-stage process: initialization, continuous topology optimization, and binarization (as shown in Figure 2). The “initialization” stage involved a random distribution of dielectric permittivity, ranging between that of silicon and silica. The “continuous topology optimization” stage comprised a forward simulation, an adjoint simulation, and perturbation. Perturbation, which serves as local optimization and correction, mitigates the adjoint method’s susceptibility to local optima. Specifically, the threshold is identified and determined based on the change in the figure of merit (FOM) between consecutive iterations. When the threshold range is exceeded, random perturbations are introduced. This approach alleviates the local-optimality defect while ensuring the normal progression of the optimization process. Following binarization, the optimized region’s dielectric permittivity distribution is converted to two discrete values of 2.0852 and 12.0873 (corresponding to “eps” in Figure 2), ensuring device manufacturability. This methodology, with the same resources and optimization configuration, can be extended to design double-layer vertical meta-grating couplers.

During the entire optimization process, three criteria for halting optimization were established: (1) the iteration count *j* reaches the predefined value *n*; (2) the figure of merit (FOM_j_) achieves the target value α; and (3) the FOM change rate ΔFOM_j_ falls within the near-zero threshold range [0,γ]. These criteria are evaluated after each forward and adjoint simulation iteration, and the optimization terminates once any single condition is met.

The introduction of random perturbations is not arbitrary but governed by threshold analysis of ΔFOM_j_. Through iterative testing, an appropriate threshold interval (θ,β) was identified to ensure that perturbation does not disrupt the optimization process (e.g., misdirecting gradient updates). The perturbation strategy, validated through multiple trials, applies small-scale global random perturbations to every pixel in the optimization domain, enabling exploration of broader parameter spaces.

## 3. Results

### 3.1. Single-Polarization Single-Layer VMGC

**Figure 3:**
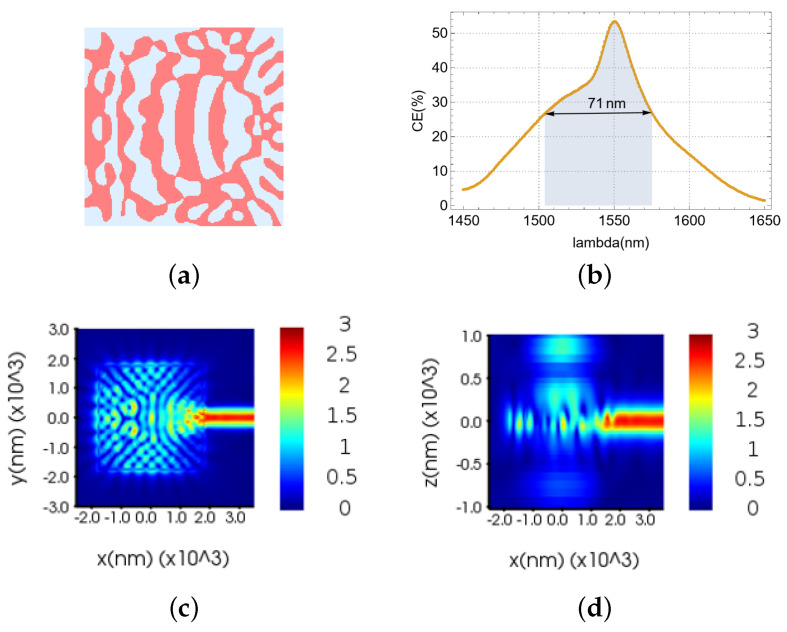
Single-polarization single-layer VMGC: (**a**) effective refractive index distribution. (**b**) CE and 3 dB bandwidth of the device. (**c**) Electric field distribution in the XOY cross-section. (**d**) Electric field distribution in the XOZ cross-section.

Initially, a fundamental single-polarization, single-layer vertical meta-grating coupler was designed, as depicted in Figure 3. The optimization domain, as illustrated in Figure 3a, is comprised solely of silicon and silica. The resultant structure exhibited notable symmetry and manufacturability. Regarding performance, Figure 3b demonstrates a coupling efficiency of 53.32% at the 1550 nm target wavelength, accompanied by a broad 3 dB bandwidth of 71 nm. The electric field distribution in the XOY plane (Figure 3c) reveals a little light leakage around the device. Furthermore, the electric field profile in the XOZ vertical plane (Figure 3d) indicates significant bottom light leakage.

### 3.2. Single-Polarization Dual-Layer VMGC

**Figure 4:**
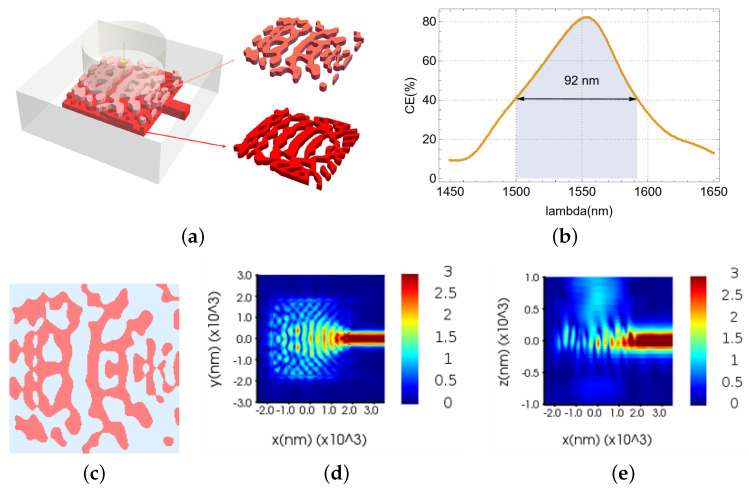
Single-polarization, dual-layer VMGC: (**a**) full 3D structure. (**b**) CE and 3 dB bandwidth of the device. (**c**) Effective refractive index distribution of the upper grating. (**d**) Electric field distribution in the XOY cross-section. (**e**) Electric field distribution in the XOZ cross-section.

Following an identical optimization procedure, the top grating of the device was optimized to achieve peak performance. The resultant structure, its performance characteristics, and electric field distributions are presented in Figure 4a–e. The device attains an 81.57% coupling efficiency and a 92 nm 3 dB bandwidth, establishing a new benchmark for VMGCs without a bottom reflector. Analysis of the electric field distributions reveals a considerable reduction in in-plane peripheral leakage (Figure 4d) and bottom leakage (Figure 4e) in the optimized double-layer meta-grating structure compared to its single-layer counterpart (Figure 3c,d).

### 3.3. Dual-Polarization Dual-Layer VMGC

**Figure 5:**
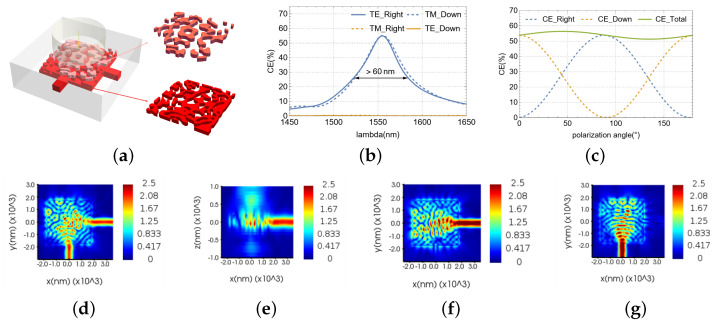
Dual-polarization dual-layer VMGC: (**a**) full 3D structure. (**b**) CE and 3dB bandwidth of the device. (**c**) PDL at 0-180°-polarized light incident at 1550nm. (**d**) E of XOY with 45° polarization source. (**e**) E of XOZ plane with 45° polarization source. (**f**) E of XOY plane with TE polarization source. (**g**) E of XOY plane with TM polarization source.

A dual-polarization, dual-layer vertical meta-grating coupler, optimized via an equivalent reverse design methodology (Figure 5a), demonstrated high efficiency and low polarization-dependent loss. The optimization targeted maximizing TE0-mode coupling efficiency at two output ports while minimizing PDL, effectively addressing polarization-induced loss. At 1550 nm, the coupler achieved 52.82% and 0.12% coupling efficiencies for the two ports under TE excitation and 0.07% and 53.11% under TM excitation. The coupler’s 3 dB bandwidth was >60 nm, with an inter-port extinction ratio > 26.4 dB (Figure 5b). Crucially, the design exhibited a PDL of <0.42 dB across all polarization states (Figure 5c). Under 45°-polarized incidence, Figure 5d,e show uniform electric field distributions at the output ports in the XOY and XOZ planes, with minimal bottom leakage. Figure 5f and Figure 5g present the field distributions with the light of TE and TM polarization, respectively.

## 4. Discussion

### 4.1. Analysis of the Key Factors Influencing the Behavior of the Optimized Device

**Figure 6:**
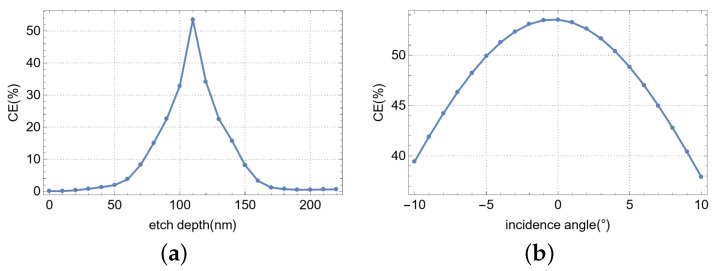
The effects of different parameters on the performance of the optimized device: (**a**) CE at various etch depths. (**b**) CE at different angles of incident light.

This study examines the influence of etch depth and incident angle on the coupling efficiency of a single-layer, single-polarization vertical meta-grating coupler. The findings, presented in Figure 6, indicate optimal coupling at an etch depth of 110 nm and an incident angle of 0°. As shown in Figure 6a, the coupling efficiency exhibits a high sensitivity to etch depth, with a 10 nm deviation inducing a 20% decline. In contrast, the device exhibits moderate robustness to variations in incident angle, as demonstrated in Figure 6b, where a 10° angular deviation results in a 17% reduction in coupling efficiency.

### 4.2. Analysis of the Effects of Optimization Parameters on Device Performance

**Figure 7:**
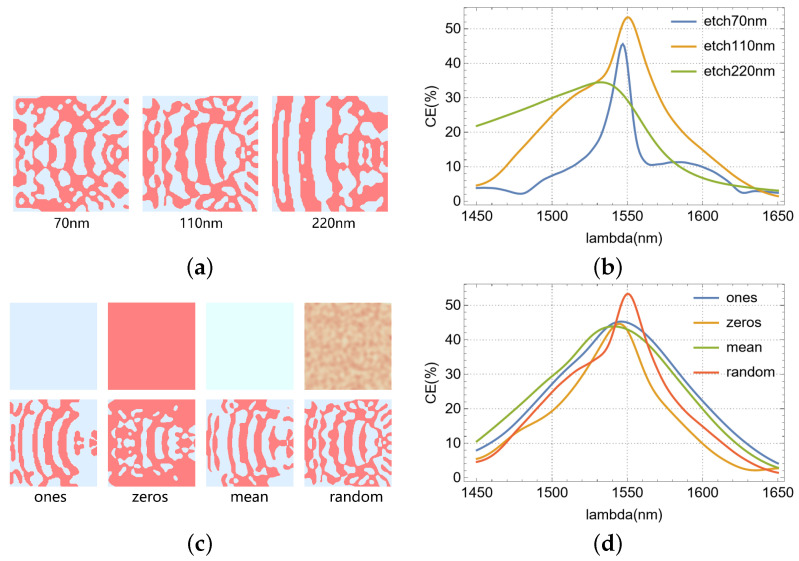
The effects of different parameters on the performance of the optimized device: (**a**) the effective refractive index distributions after optimization with different etching depths. (**b**) CE of devices with different etch depths. (**c**) The effective refractive index distributions after optimization with different initial structures. (**d**) CE of the device with diverse initial structure.

We analyzed the impact of etch depth on optimization results, which are visualized in Figure 7a,b. Figure 7a shows the effective refractive index distributions of the completely different final optimized structures obtained when the initial parameter of “etching depth” is set to different values. Examination of Figure 7b reveals a positive correlation between the 3 dB bandwidth of the optimized devices and the etch depth, considered at full (220 nm), half (110 nm), and shallow (70 nm) depths. The highest coupling efficiency was observed when the grating was half-etched (110 nm). The choice of initial structure is critical for the optimization outcome. Different initial conditions invariably lead to variations in both optimized structure morphology and device performance. The half-etching depth of 110 nm effectively balances scattering strength and radiation loss, achieving higher coupling efficiency than shallow etching (70 nm) and full etching (220 nm). Figure 7c illustrates four initial refractive index distributions (corresponding to uniform SiO2(ones), uniform Si(zeros), the mean effective index of Si and SiO2(mean), and a random distribution within their range(random)) and their respective optimized counterparts. Notably, optimization with a randomized initial effective refractive index distribution yielded the highest coupling efficiency, as demonstrated in Figure 7d. This is because the randomized initial refractive index distribution outperforms the uniform distribution as it enables the optimization algorithm to escape local extrema and explore a broader design space. While uniform distributions restrict gradient optimization paths, randomness aids in discovering globally efficient configurations.

### 4.3. Fabrication Process

We aim for seamless device compatibility with existing silicon manufacturing. Two steps are taken: smoothing grating edges and adjusting tiny structures (e.g., silicon blocks, holes) smaller than the min. feature size to meet standards. Research shows that, for our 110 nm etching, the stable accuracy min. feature size is 40 nm, and our devices meet this. Despite this edge rounding, our single-polarization, single-layer perpendicular meta-grating coupler maintains a simulated coupling efficiency exceeding 53%. Given the existing silicon fabrication process with a 40 nm feature size at a 110 nm etch depth, we modified the sawtooth edge dimension from 20 nm to 40 nm. This adjustment resulted in a simulated coupling efficiency of approximately 51%.

Regarding dual-layer grating fabrication, three primary approaches exist: (1) direct silicon growth and etching on the lower grating, (2) separate etching and subsequent alignment/bonding, and (3) lower grating polishing followed by growth and etching of the upper layer. The first method risks damaging the underlying structure, the second demands high alignment precision, and the third necessitates an intermediate isolation layer. Despite the current challenges in the fabrication process, it is foreseeable that our structure can be fabricated using these methods, and the performance variations introduced by different processing techniques are tolerable.

**Figure 8:**
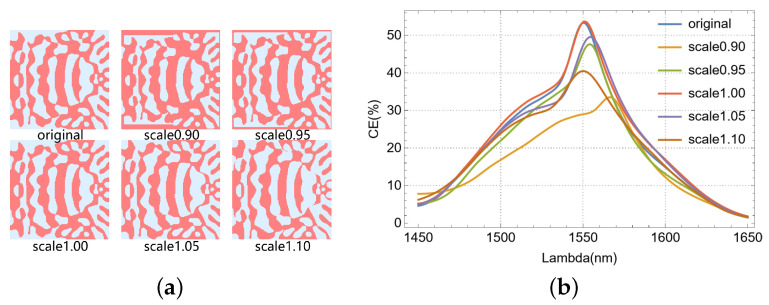
Process robustness analysis of the device: (**a**) effective refractive index distribution of the corresponding structure at different scale factors. (**b**) CE of the device with different scale factors.

Furthermore, we investigated the processing robustness of the device performance, as illustrated in Figure 8. Specifically, by applying scaling factors ranging from 0.90 to 1.10 in 0.05 increments to the etch hole dimensions, we observed a concurrent reduction in overall peak coupling efficiency. This efficiency degradation exhibited a positive correlation with the magnitude of the scaling factor’s deviation from unity. Figure 8a depicts the refractive index profiles of the optimized region under varying etch error conditions. As shown in Figure 8b, the coupling efficiency of our single-polarization, single-layer VMGC decreases by less than 8% at a ±5 nm etch error. While a 10 nm over-etch still resulted in the coupling efficiency exceeding 40%, a 10 nm under-etch significantly reduced efficiency by almost half. This significant reduction stems from large errors in our dimensional scaling method during under-etching, as it notably affects the edge scaling of multi-connected vias. As we understand, electron-beam etching equipment has a nominal accuracy of ±5%. For a 40 nm minimum feature size, the ±2 nm etching error negligibly impacts our device’s performance. Thus, our designed device shows high process robustness.

### 4.4. Selection of Materials

In terms of etched holes and cladding material selection, air represents a common alternative to the silica employed in this study. The performance of devices fabricated using air as the etched-hole material is comparable to that of devices using silica. Air is easier to work with in the manufacturing process, yet the devices are more susceptible to damage. Furthermore, during the design phase, silicon nitride was explored as a potential material for the upper grating structure. Compared to the performance levels demonstrated in this investigation, silicon nitride exhibited an approximately 18% reduction in coupling efficiency and a 13 nm bandwidth shrinkage. It is important to note that the design of these alternative materials must be compatible with their existing fabrication processes.

### 4.5. Optimization Efficiency and Effectiveness

In this work, all device optimizations were conducted on a computer equipped with an Intel Xeon Platinum 8352 V CPU and 1 TB of memory. Taking the single-polarization, single-layer VMGC as an example, our method outperformed Lumopt in optimization efficiency. It took 189 iterations and approximately 15.75 h, while Lumopt needed 488 generations and around 46.6 h under identical parameters, indicating a nearly three-fold enhancement in optimization speed.

**Figure 9:**
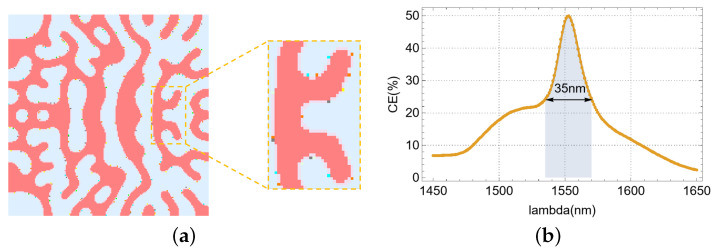
The results of the optimized device with Lumopt: (**a**) the effective refractive index distribution. (**b**) CE and 3dB bandwidth of the device.

In terms of optimization effectiveness, devices optimized using Lumopt exhibited structures that were not fully binarized into silicon and silicon dioxide, unlike those obtained with our method. Furthermore, they also demonstrated inferior coupling efficiency and 3 dB bandwidth, as detailed in Figure 9a,b, respectively. Nevertheless, it must be acknowledged that the Lumopt tool demonstrates a superior filtering effect throughout the optimization process. Virtually no silicon blocks or etched holes smaller than the filtering radius are present, thereby imposing less stringent requirements on the processing technology. We attempted to substitute the materials between silicon and silica with either silicon or silica via a simple threshold treatment. However, this led to more significant performance degradation.

### 4.6. About MFD

Regarding the mode field diameter, due to our compact size of 3.6 μm × 3.6 μm, it is impossible to effectively capture all the energy of a standard optical fiber with an MFD of 10.4 μm ± 0.5 μm in the C-band. For instance, under a mode field diameter of 10.4 μm, our designed single-polarization bilayer vertical grating coupler exhibited coupling efficiency and 3dB bandwidth reduced to 51.08% and 82 nm, respectively, which were deemed non-substantive for practical applications. Therefore, in practical applications, we consider using tapered optical fibers to convert the mode field diameter of the standard optical fiber to the smaller one that we need with low loss so as to achieve the expected coupling efficiency.

### 4.7. Versatility and Scalability of the Improved ADJ

The improved ADJ proposed in this study exhibits broad applicability across diverse material platforms and on-chip passive photonic devices. These platforms encompass silicon, silicon nitride, lithium niobate, and even multi-layered or hybrid material systems. For passive photonic components such as wavelength (de)multiplexers, polarization (de)multiplexers, waveguide crossings, power dividers, and grating couplers, the method requires only three steps to initiate inverse design workflows: (1) constructing a complete initial geometry, (2) defining light sources and monitoring regions, and (3) specifying simulation parameters.

Notably, optimization objectives may vary significantly between device types. This challenge is addressed by customizing the objective function within the proposed framework to align with specific performance requirements. The demonstrated flexibility highlights the method’s high generality and scalability, underscoring its promising potential for advanced photonic integrated circuit design.

### 4.8. About Back-Reflections

**Figure 10:**
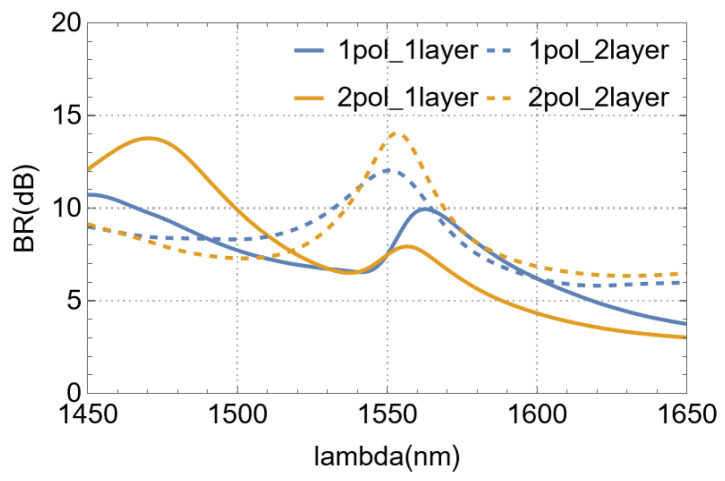
The results of devices’ back-reflection.

For VMGCs, back-reflection is one of the main factors affecting coupling efficiency. Figure 10 compares the back-reflection(BR) performance of single-polarization and polarization-splitting devices with single-layer and dual-layer structures. At the wavelength of 1550 nm, compared with the single-layer structure, the dual-layer structure increases the back-reflection suppression by 4.5 dB for the single-polarization device and approximately 6.23 dB for the polarization-splitting device, which verifies the superiority of our dual-layer design in suppressing back-reflection.

However, it is worthy of reflection that the back-reflection of the two current double-layer devices is around 13 dB, which can only meet the signal transmission requirements of ordinary optical systems. For high-speed, long-distance optical communication systems or optical precision measurement systems with extremely high requirements for signal stability, the performance still needs to be improved.

### 4.9. Literature Review

This review examines the recent literature on vertical grating couplers, focusing on publications from the past five years, as summarized in Table 1. The device presented in this study showcases a more compact footprint compared to traditional grating coupler designs. Notably, in the context of inverse-designed vertical grating couplers without a bottom reflector, our device achieves superior coupling efficiency and broader bandwidth. Therefore, the vertical meta-grating coupler developed in this work provides significant advancements.

## 5. Conclusions

In this paper, we have presented an improved topology optimization methodology. This innovative technique effectively accelerates the optimization process while incorporating random perturbations to mitigate the limitations of local optima. Utilizing this method, we successfully designed and demonstrated high-efficiency, ultra-compact VMGCs for both single- and dual-polarization operations on the standard 220 nm SOI platform. These devices achieve a minimal footprint of just 3.6 μm × 3.6 μm, demonstrating considerable potential for applications requiring miniaturized optical components.

Our designed single-polarization VMGC demonstrated a peak coupling efficiency of 81.57% and a 3 dB bandwidth of 92 nm. This represents, to the best of our knowledge, the highest reported performance for such a device without the incorporation of a bottom reflector. For the dual-polarization operation, the coupler achieved coupling efficiencies of 52.82% and 53.11% for transverse electric (TE) and transverse magnetic (TM) polarizations, respectively. These results were accompanied by a 3 dB bandwidth exceeding 60 nm, an ER exceeding 26.4 dB, and a PDL below 0.42 dB.

A systematic analysis was undertaken to assess the impact of etching depth, light incidence angle, initial structure, material, MFD, and fabrication process on device performance and optimization effectiveness. This study provides key insights crucial for the practical design and optimization of related devices. The developed methodology exhibits general applicability for the design of meta-photonic devices. The designed grating coupler is a significant contribution to large-scale meta-photonic integrated circuits, offering potential advancements in photonic integrated circuit lidar, optical communications, optical imaging, inverse design of photonic devices, and others.

## Figures and Tables

**Table 1:** A comparison of studies of VMGCs on the 220 nm SOI platform.

Refs./Year	Design Method and Tools	Special Features	Taper	Polarization	Size of GC (μm)	CE (%)	BW (nm)
[20]/2018	Shape optimization method	Dual-layer	No	single	∼15	96.9	24 ^*a*^
[21]/2022	Forward design method + PSO	/	Yes	single	∼200 × 14	84.14	47 ^*a*^
[13]/2022	Neural networks	Multiple etch depth	Yes	single	14 × 14	53.10	∼70 ^*b*^
		/	Yes	dual	14 × 14	22.39 /25.40	68 ^*b*^ /79 ^*b*^
[22]/2021	Lumos	/	No	single	2.8 × 2.8	32.45	103 ^*b*^
		Bottom reflector	No	single	2.8×2.8	88.42	103 ^*b*^
[9]/2024	Lumos	Bottom reflector	No	single	2.8×2.8	77.98	128 ^*b*^
[10]/2024	ADJ and Lumopt	Bottom reflector	No	single	14×14	92.2	35 ^*b*^
[12]/2022	ADJ+GCMMA	Dual-layer	No	single	10 × 10	50.12	73 ^*b*^
		Dual-layer	No	dual	10 × 10	27.54	/
This work	Improved ADJ	/	No	single	3.6 × 3.6	53.32	71 ^*b*^
		Dual-layer	No	single	3.6 × 3.6	81.57	92 ^*b*^
		Dual-layer	No	dual	3.6 × 3.6	52.82 /53.11	>60 ^*b*^

^*a*^ is 1 dB bandwidth; ^*b*^ is 3 dB bandwidth; GC: grating coupler; CE: coupling efficiency; BW: bandwidth.

## Data Availability

The data presented in this study are available on reasonable request from the corresponding author.

## References

[1] Li C., Wu K., Cao X., Zhang G., Li T., Deng Z., Chang M., Wang Y., Li X., Chen J. (2022). Monolithic coherent LABS lidar based on an integrated transceiver array. Opt. Lett..

[2] Zhang X., Kwon K., Henriksson J., Luo J., Wu M.C. (2022). A large-scale microelectromechanical-systems-based silicon photonics LiDAR. Nature.

[3] Cheng Q., Bahadori M., Glick M., Rumley S., Bergman K. (2018). Recent advances in optical technologies for data centers: A review. Optica.

[4] Rogers C., Piggott A.Y., Thomson D.J., Wiser R.F., Opris I.E., Fortune S.A., Compston A.J., Gondarenko A., Meng F., Chen X. (2021). A universal 3D imaging sensor on a silicon photonics platform. Nature.

[5] Sun J., Timurdogan E., Yaacobi A., Hosseini E.S., Watts M.R. (2013). Large-scale nanophotonic phased array. Nature.

[6] Cheng L., Mao S., Li Z., Han Y., Fu H. (2020). Grating couplers on silicon photonics: Design principles, emerging trends and practical issues. Micromachines.

[7] Zhang X., Kwon K., Henriksson J., Luo J., Wu M.C. A 20 × 20 focal plane switch array for optical beam steering. Proceedings of the 2020 IEEE Conference on Lasers and Electro-Optics (CLEO).

[8] Zhang J., Yang J., Xin H., Huang J., Chen D., Zhaojian Z. (2017). Ultrashort and efficient adiabatic waveguide taper based on thin flat focusing lenses. Opt. Express.

[9] Wang L., Qiu J., Dong Z., Chen Y., Wu L., Guo H., Wu J. (2024). Single-etched fiber-chip coupler with a metal mirror on a 220-nm silicon-on-insulator platform for perfectly vertical coupling. Opt. Lett..

[10] Huang S.Y., Barz S. (2025). Compact inverse designed vertical coupler with bottom reflector for sub-decibel fiber-to-chip coupling on silicon on insulator platform. Sci. Rep..

[11] Notaros J., Popović M.A. Band-structure approach to synthesis of grating couplers with ultra-high coupling efficiency and directivity. Proceedings of the Optical Fiber Communication Conference.

[12] Hammond A.M., Slaby J.B., Probst M.J., Ralph S.E. (2022). Multi-layer inverse design of vertical grating couplers for high-density, commercial foundry interconnects. Opt. Express.

[13] Liu C. (2022). Inverse Design of Micro—Nano Photonic Devices Based on Neural Network. Master’s Thesis.

[14] Kang S., Gao F., Yu X., Bo F., Zhang G., Xu J. (2023). Lithium niobate thin film grating couplers optimized by particle swarm optimization and a neural network. JOSA B.

[15] Meng Y., Chen Y., Lu L., Ding Y., Cusano A., Fan J.A., Hu Q., Wang K., Xie Z., Liu Z. (2021). Optical meta-waveguides for integrated photonics and beyond. Light. Sci. Appl..

[16] Cheben P., Halir R., Schmid J.H., Atwater H.A., Smith D.R. (2018). Subwavelength integrated photonics. Nature.

[17] Zhu L., Yang W., Chang-Hasnain C. (2017). Very high efficiency optical coupler for silicon nanophotonic waveguide and single mode optical fiber. Opt. Express.

[18] Li Z., Zhou Z., Qiu C., Chen Y., Liang B., Wang Y., Liang L., Lei Y., Song Y., Jia P. (2024). The Intelligent Design of Silicon Photonic Devices. Adv. Opt. Mater..

[19] Chen Y., Yuan H., Zhang Z., Ma H., Chen J., Li X., Yang J. (2023). Design of multi-wavelength demultiplexing coupler based on DBS algorithm. Opt. Commun..

[20] Michaels A., Yablonovitch E. (2018). Inverse design of near unity efficiency perfectly vertical grating couplers. Opt. Express.

[21] Liu J., Zheng Z., Chen B., Wang Z., Li C., Chen K., Liu L. (2022). High-performance grating coupler array on silicon for a perfectly-vertically mounted multicore fiber. J. Light. Technol..

[22] Chen Y., Qiu J., Dong Z., Pan Y., Chen Y., Guo H., Wu J. (2021). Inverse Design and Demonstration of Vertical Couplers. Acta Opt. Sin..

